# Validity of self-reported concentration and memory problems: Relationship with neuropsychological assessment and depression

**DOI:** 10.1080/13803395.2017.1301392

**Published:** 2017-03-29

**Authors:** Rosemarie M. Bowler, Shane W. Adams, Ralf Schwarzer, Vihra V. Gocheva, Harry A. Roels, Yangho Kim, Catherine L. Kircos, Chris W. Wright, Michelle Colledge, George Bollweg, Danelle T. Lobdell

**Affiliations:** a Department of Psychology, San Francisco State University, San Francisco, CA, USA; b Institute for Positive Psychology and Education, Faculty of Health Sciences, Australian Catholic University, Strathfield, NSW, Australia; c Department of Clinical, Health, and Rehabilitation Psychology, SWPS University of Social Sciences and Humanities, Warsaw, Poland; d Louvain Centre for Toxicology and Applied Pharmacology (LTAP), Université catholique de Louvain, Brussels, Belgium; e Department of Occupational and Environmental Medicine, Ulsan University Hospital, University of Ulsan College of Medicine, Ulsan, South Korea; f ATSDR, Chicago, IL, USA; g U.S. EPA, Chicago, IL, USA; h U.S. EPA, National Health and Environmental Effects Research Laboratory, Research Triangle Park, NC, USA

**Keywords:** Cognitive, manganese, neuropsychology, self-report, validity

## Abstract

**Background:**

This study investigated the validity of self-reported concentration and memory problems (CMP) in residents environmentally exposed to manganese (Mn).

**Method:**

Self-report of CMP from a health questionnaire (HQ) and the Symptom Checklist-90-Revised (SCL-90-R) was compared to neuropsychological assessment (Trails A&B; Digit Span; Digit Symbol; Similarities; Auditory Consonant Trigrams, ACT; NAB Memory; Rey–Osterrieth, Rey–O, Delayed). Participants included 146 residents from Ohio exposed to air-Mn, with a modeled average concentration of 0.55 μgm^−3^ (range = 0.01–4.58).

**Results:**

Residents were primarily White (94.5%), aged 30–64 years (*M* = 51.24), with a minimum of 10 years of residence (range = 10–64). Ninety-four (65.3%) participants reported concentration problems, and 107 residents (73.3%) reported memory problems. More participants endorsed CMP on the SCL-90-R than on the HQ. The prevalence of self-reported CMP was higher for women than for men (88.4% vs. 68.3%). Point-biserial and Pearson's correlations between self-reported CMP and neuropsychological test scores were nonsignificant and weak for both the HQ (*r*_pb_ = −.20 to *r*_pb_ = .04) and the SCL-90-R (*r* = −.12 to r = .007). Greater levels of depression, anxiety, and female sex predicted having more self-reported CMP on both the HQ and the SCL-90-R. Air-Mn and blood-Mn were not associated with self-reported CMP. Residential distance from the Mn source accounted for a small proportion of variance (*sr*^2^ = .04), although depression remained the largest predictor (*sr*^2^ = .21).

**Conclusion:**

These results indicate that self-report of CMP in Mn-exposed residents appear to be invalid when compared to neuropsychological test scores. The participants' misperception of having CMP is associated with less education and higher levels of depression. Neuropsychological assessment is recommended to attain valid results.

Concentration and memory problems are some of the most common complaints presented to clinical neuropsychologists (Lezak, Howieson, Bigler, & Tranel, 2012; McConnell & Crockett, 1994). Assessing the validity of self-reported concentration and memory problems is required when determining the real-world utility of such reports. Although self-reported concentration and memory problems are frequently used in epidemiological surveys and may provide a simple and straightforward way to screen for these cognitive problems, it is not clear whether self-report of cognition is well correlated with clinical neuropsychological test performance. Furthermore, findings from the literature are primarily limited to older populations (Crumley, Stetler, & Horhota, 2014). It has been suggested that individuals with memory impairment, as determined by clinical assessment, may misperceive their problems or, may forget about their memory failures, potentially rendering their cognitive self-report invalid (Herrmann, 1982). Differences in level of insight or self-perception can also result in the overreporting of concentration and memory symptoms, for example in clinically depressed patients (Lezak et al., 2012).

## Studies of self-report items with neuropsychological test scores

The majority of studies investigating the relationship between self-reported cognitive problems and neuropsychological test performance have been conducted primarily in older adults. Most of the studies report either small or nonsignificant relationships between self-report and neuropsychological test performance (Bast-Pettersen, 2006; Jungwirth et al., 2004; McConnell & Crockett, 1994; Uttl & Kibreab, 2011). Other studies have found statistically significant but still very weak associations between self-report of cognitive problems and objective test performance in certain domains of cognitive function, such as episodic memory, remote memory, executive functioning, and visuospatial and verbal memory (Chin, Oh, Seo, & Na, 2014; Langlois & Belleville, 2014; Larrabee & Levin, 1986; Lenehan, Klekociuk, & Summers, 2012; Volz-Sidiropoulou & Gauggel, 2012). Crumley et al. (2014) conducted a meta-analysis of 53 studies of older cognitively “normal” adults (age range = 60–81 years) to investigate the association between self-reported memory and objective memory performance. They determined that subjective memory problems accounted for less than 1% of the variance in objective memory performance. Overall, previous findings indicate that self-reports of concentration and memory problems are questionable indicators of objective cognitive function.

Demographic factors (e.g., age, sex, education) must be considered when examining correlations between subjective and objective measures of concentration and memory problems. In a review of the topic, Iliffe and Pealing (2010) found that more subjective memory problems were associated with older age and female sex. The strength of the correlation between subjective and objective memory has been suggested to increase positively with age (Crumley et al., 2014; Parisi et al., 2011). Additionally, individuals with higher levels of education appear to self-report their cognitive abilities more accurately, whereas lower education is associated with increased self-report of memory problems (Crumley et al., 2014; Genziani et al., 2013; Hülür, Hertzog, Pearman, Ram, & Gerstorf, 2014).

## Cognition and manganese

Manganese (Mn) is an essential metal that, at excessive exposure, may show neurotoxic properties associated with cognitive (Bast-Pettersen, Ellingsen, Hetland, & Thomassen, 2004; Bowler et al., 2015; Chang et al., 2010; Mergler et al., 1999; Roels et al., 2012; Solís-Vivanco et al., 2009) and movement disorder symptoms (Bowler, Adams, et al., 2016; Kim et al., 2011; Lucchini, Bergamaschi, Smargiassi, Festa, & Apstoli, 1997; Rodriguez-Agudelo et al., 2006; Roels, Ghyselen, Buchet, Ceulemans, & Lauwerys, 1992; Roels et al., 1987). For adults, the majority of reports of the health effects of Mn are from occupational studies, which typically have higher levels of Mn in air than in environmental studies. In environmental studies of Mn exposure, elevated levels of Mn in air (air-Mn) have been associated with decreased attention and concentration, learning, abilities, verbal memory, and visuospatial memory (Bowler et al., 2015; Mergler et al., 1999; Santos-Burgoa et al., 2001).

A previous study by the authors (Bowler et al., 2015) examined cognitive function in residents of two Ohio towns examined in this study (Marietta and East Liverpool) environmentally exposed to air-Mn with a modeled average concentration of 0.55 μg m^−3^ (range = 0.01–4.58). All residents were administered an extensive neuropsychological test battery including tests of concentration and memory. Significant negative associations were found between modeled air-Mn concentrations and measures of visuospatial memory, daily living memory, and verbal skills.

The objective of this study was to expand upon the current literature reporting the validity of self-reported cognitive complaints in populations of older adults, to assess these problems in an exposed middle-aged community sample using an extensive neuropsychological test battery, and, if cognitive complaints are misperceived, to identify factors that predict this misperception. To accomplish this, we examined the relationship between self-reported or perceived concentration and memory problems as compared to clinical neuropsychological test scores in a group of middle-aged residents from two Ohio towns environmentally exposed to Mn. In addition, both dichotomized and scaled self-report metrics were analyzed to assess the validity of multiple self-report measures.

## Method

### Study design and participant selection

This study is part of a larger cross-sectional study assessing the neurotoxic effects of air-Mn in residents living near a ferromanganese smelter in Marietta, Ohio, and an open-air Mn storage and packaging facility in East Liverpool, Ohio (Bowler, Adams, et al., 2016; Bowler, Beseler, et al., 2016; Bowler et al., 2012; Kim et al., 2011). Both Marietta and East Liverpool have been reported by the U.S. Environmental Protection Agency (EPA) to have some of the highest measured environmental concentrations of respirable air-Mn in the U.S. (U.S. EPA, 2011 U.S. EPA, 2012). For Marietta residents, a random sample of parcels was drawn from December 2008 property tax records within the predefined Mn exposure zone of 0.04 μg m^−3^ or higher (12-mile distance range). In East Liverpool, a sample of addresses located within two miles of the point-emission source was purchased from a commercial vendor (Spectrum Mailing Lists) in 2011. This list was crosschecked with East Liverpool parcel maps and the Columbiana County emergency response database and, then processed through Geographic Information Systems (GIS) mapping at the Agency for Toxic Substances and Disease Registry (ATSDR). In Marietta, 1732 letters were mailed to randomly selected addresses and were successfully delivered to 1569 residents of which 264 residents were interested, 122 were eligible for participation, and 100 were tested. In East Liverpool, 1309 letters were mailed, of which 1213 were successfully delivered, 192 residents were interested, 123 were eligible for participation, and 86 were tested (14 residents did not attend their appointment). The recruitment letters and consent forms indicated that the purpose of the study was to investigate potential health effects of Mn exposure.

The inclusion and exclusion criteria for Marietta and East Liverpool were identical. A minimum of 10 years of residency in the respective towns was required. Other inclusion criteria in the prior study (Bowler et al., 2012) included: participants aged 30–75 years (note next paragraph) without a major illness or exposure to toxic substances requiring hospitalization, without a medical diagnosis of psychiatric (schizophrenia, bipolar disorder, major psychiatric diagnosis, including depression or anxiety) or degenerative (multiple sclerosis, Alzheimer's dementia, Huntington's chorea, Parkinson's disease) disorder, and never having worked at either the Mn-emitting smelter facility or the Mn-alloy storage and processing plant. Up to two members of a household could participate. Participants were given $50 gift certificates upon completion of the testing.

The present study restricted the age to between 30 and 64 years at the time of testing because of reported differences in older populations in mood and increased self-report of cognitive problems (Crumley et al., 2014; Iliffe & Pealing, 2010; Parisi et al., 2011). Thus, 146 residents, 83 (56.8%) from Marietta and 63 (43.2%) from East Liverpool, were included. When compared with 2008–2012 U.S. Census Bureau data, study participants from Marietta and East Liverpool were found to be mostly representative (with the exception of education) of the two respective towns (Bowler, Beseler, et al., 2016).

The identical Marietta and East Liverpool study protocols were approved by the Institutional Review Boards at San Francisco State University, the Ohio Department of Health, and the University of North Carolina at Chapel Hill on behalf of the U.S. EPA.

### Procedure and study materials

Data were collected in August 2009 (Marietta) and November 2011 (East Liverpool) at a central location in each town. Participants completed the health questionnaire (HQ) as well as a psychiatric symptom inventory, the Symptom Checklist-90-Revised (SCL-90-R; Derogatis, 1992). Both the HQ and the SCL-90-R include items that ask participants about having concentration and memory problems. Consequently, the present study used the HQ as a dichotomized report and SCL-90-R as a scaled report of self-reported concentration and memory problems. Participants from each town completed the HQ and all neuropsychological testing, as well as personal interviews with a neuropsychologist.

### Health questionnaire

The HQ included two dichotomized items (“Yes” or “No”) that ask the participant if they are experiencing any symptoms of having “difficulty concentrating” or “trouble remembering things.” The SCL-90-R includes two scaled items that ask the participant to rate “how much [the] problem has distressed or bothered [them] during the past 7 days including today” (Derogatis, 1992). The two scaled items included in this study are “trouble concentrating” and “trouble remembering things,” which are measured on a 5-point scale (0 = *not at all*, 1 = *a little bit*, 2 = *moderately*, 3 = *quite a bit*, 4 = *extremely*).

### SCL-90-R

The 90 items of the SCL-90-R are grouped into nine subscales and three global indices. The two scaled items from the SCL-90-R measuring concentration and memory problems are part of the Obsessive-Compulsive symptom scale. Levels of depression and anxiety in the present study were measured using the Depression and Anxiety symptom scales, which are designed to provide the examiner with a description of the participant's mood symptoms and their intensity. The scores are standardized using sex-adjusted norms. The SCL-90-R Depression scale consists of 13 questions representing the range of symptoms of clinical depression, including dysphoric mood, anhedonia and lack of motivation, suicidal thoughts, and excessive worry. The Anxiety scale includes 10 symptoms associated with increased clinical levels of anxiety, such as symptoms of nervousness, panic, trembling, apprehension, and dread. Both scales have excellent internal consistency (α = .90 for the Depression scale and α = .85 for the Anxiety scale) and test-retest reliability (.82 and .80, respectively; Derogatis, 1992).

The SCL-90-R differs from the health questionnaire and other self-report measures because it is a standardized and normed self-report inventory extensively used in a wide array of populations (Ransom, Ashton, Windover, & Heinberg, 2010; Schmitz, Kruse, Heckrath, Alberti, & Tress, 1999) as a tool for psychological screening and treatment effectiveness monitoring. In addition to having high clinical utility, the SCL-90-R has been used in many, health evaluations and studies of neuropsychological disorders, including neurotoxic exposures (Lezak et al., 2012).

### Tests of concentration and memory

Administered as part of a larger cognitive test battery, tests of concentration and memory were chosen to validate the self-report of concentration and memory problems. Tests used to objectively assess concentration include the Trail Making Test A and B (Trails A and B; Heaton, Miller, Taylor, & Grant, 2004), Wechsler Adult Intelligence Scale–III (WAIS–III) Digit Span, WAIS–III Digit Symbol Coding, and WAIS–III Similarities (Wechsler, 1997). Tests used to objectively measure memory performance included the Auditory Consonant Trigrams (ACT; Boone, Miller, Lesser, Hill, & D'Elia, 1990), NAB Memory Index (Stern & White, 2003), and Rey–Osterrieth (Rey–O) Delayed Recall (Strauss, Sherman, & Spreen, 2006). The order of test administration was consistent for all participants, and tests were administered by experienced psychometricians with advanced degrees in psychology in accordance with standardized administration instructions published by the respective test publishers. Table 1 displays the neuropsychological tests by domain of function and type of score.

### Effort

The Rey 15-Item Memory Test (Lezak et al., 2012) was administered to all participants to screen for potential memory malingering. A conservative cutoff point of ≤8 was applied, and the Victoria Symptom Validity Test (VSVT; Slick, Hopp, Strauss, & Thompson, 1997) was administered to participants scoring below the cut-point. In this study sample, only one participant scored below the Rey 15-Item cutoff, consequently obtaining perfect scores on the VSVT and valid test scores. All tests were administered by experienced psychometricians who were extensively trained by the principal investigator (PI) in obtaining best performance from participants. To our knowledge, none of the participants were involved in compensation-seeking litigation or procedures at the time of data collection, and to date none have requested their data for litigation or other purposes.

### Mn exposure estimates

Colledge et al. (2015) described in detail the personal residential outdoor exposure estimates of airborne Mn and the methodology used to develop the estimates. Briefly, air concentrations from the Mn point source facilities, participant residences, and air monitoring sites were modeled using the U.S. EPA's AERMOD dispersion model, using an assumed unit emission rate of 1 g s^−1^ over the surface area. A long-term air monitor was used as a reference location for the three area monitors, and ratios of all modeled receptor points to that monitor were computed using air measurements from the reference location (Bowler et al., 2015; Colledge et al., 2015). Exposures were assumed to be long term given that inclusion criteria included residence of ≥10 years. Air sampling in both towns was performed over 10 years from 2003–2013 when sampling data met 75% completeness in both towns (Colledge et al., 2015). Sampling and analytical methods were identical for both towns. Modeled all-year average air-Mn (TSP: total suspended particulate) exposure in the environment ranged from 0.03 to 1.61 μg m ^−3^ (*M* = 0.21 μg m^−3^ ) in Marietta and 0.01–6.32 μg m ^−3^ (*M* = 0.88 μg m ^−3^) in East Liverpool.

Recruitment zones were based on estimated “impact radius” for each town from the Mn point source. Estimates were provided by the U.S. EPA and the National Enforcement Investigations Center (NEIC). The ATSDR concluded that Mn was the only metal exceeding background levels and government guidelines (ATSDR, 2009, 2010).

### Statistical analyses

Exploratory data analyses revealed no outliers as measured by Cook's *d*. Pearson's product–moment, coefficient and point-biserial correlation were used to examine the convergent validity between the scaled and dichotomous concentration and memory items and neuropsychological test scores, respectively. Due to the number of bivariate correlations examined, adjustments for multiple comparisons were made using the Benjamini-Hochberg false discovery rate to provide a more conservative null hypothesis test (Benjamini & Hochberg, 1995). All bivariate correlation results incorporated adjusted *p*-values, which reflect the q* value after adjusting for multiple comparisons. Medium effect sizes may be considered clinically relevant (Cohen, 1992). To examine predictors of scaled and dichotomous responses to concentration and memory items, hierarchical linear (scaled) and logistic (dichotomous) regressions were used.

Collinearity was found (*r* ≥ .70; variance inflation factor, VIF ≥ 2.4) when examining SCL-90-R Depression and Anxiety *T* scores in the multiple regression models. When such collinearity is present, orthogonalization through residualization is suggested to better assess the independent contributions of each predictor to the multiple regression models (Baayen, 2008; Geldhof, Pornprasertmanit, Schoemann, & Little, 2013; Kuperman, Bertram, & Baayen, 2008). Because the literature suggests that depression is the main predictor of self-reported concentration and memory problems (Chin et al., 2014; Iliffe & Pealing, 2010; Langlois & Belleville, 2014; McConnell & Crockett, 1994), we residualized the variable of anxiety. This ensures that the variable of anxiety represents only the unique variance that anxiety contributes to the model, independent of depression. Orthogonalization was achieved, and no other cases of collinearity were present. The variable of “residualized anxiety” was used for all multiple regression models.

## Results

Table 2 shows the sociodemographics, exposure, and cognitive complaints for the two towns combined. Participants (*n* = 146) were predominantly White (94.5%), with a majority of women (58.9%), and a mean age of 51.2 years (range = 30–64). On average, participants lived in their respective towns for 38.6 years and had 13.9 years of education. Air-Mn site surface emissions method modeling for TSP ranged from 0.01 to 4.58 μg m^−3^ with a mean of 0.55 μg m^−3^.

More participants reported concentration (64.4% vs. 27.1%) or memory (71.9% vs. 45.2%) problems using a scaled response (SCL-90-R) than a dichotomized response (HQ), respectively. Overall, more participants reported concentration problems than memory problems.

All bivariate correlations (Table 3) between self-reported concentration and memory problems and neuropsychological test scores were nonsignificant and small for both the dichotomized (*r*_pb_ = −.20 to *r*_pb_ = .04) and scaled items (*r* = −.12 to *r* = .007).

### Regression analyses predicting self-reported concentration or memory problems

To determine whether anxiety and/or depression predicted self-reported concentration or memory problems using the dichotomized (Table 4) and scaled items (Table 5), further analyses were conducted using binomial logistic regression and hierarchical multiple linear regression, respectively. Age, sex, years of education, and household income were entered as covariates in Step 1 of the regression model, and depression and anxiety scores were entered in Step 2. Other psychiatric symptom scales from the SCL-90-R (e.g., Somatization) were included in the original regression model. However, these variables did not significantly contribute to the overall model when equally considered with covariates and Depression and Anxiety subscale scores. Therefore, they were excluded from the final model.

When examining dichotomized self-report of concentration problems, higher levels of depression were the only significant predictor (Table 4). When examining dichotomized self-report of memory problems, higher levels of depression, female sex, and fewer years of education were significant predictors.

When predicting scaled reports of concentration and memory problems, models for both concentration (*R*^2^_adj_ = .42) and memory (*R*^2^_adj_ = .25) problems showed good model fit and overall effect (Table 5). Higher levels of depression accounted for the largest proportion of total variance for the scaled response in the self-report of concentration (*sr*^2^ = .39) and memory problems (*sr*^2^ = .20) over and above the other predictors. Higher levels of anxiety and female sex were weak predictors of increased self-report of concentration problems. Analyses using the scaled items demonstrated greater measured effects than those using the dichotomous items.

### Differences between men and women

A higher proportion of women self-reported both concentration (76.2% vs. 50.0%), χ^2^ = 10.59, p = .001, and memory (79.1% vs. 65.0%), χ^2^ = 3.57, p = .045, problems than men. To investigate why more women had more reported concentration and memory problems than men, the same regression model was stratified by sex. The new model was a better fit for men (*R*^2^_adj_ = .55) than for women (*R*^2^_adj_ = .28), indicating that there might be a variable not included in the model that affects the self-perception of concentration and memory problems in women. Higher levels of depression accounted for over half of the variation in self-reported concentration problems for men (*sr*^2^ = .51), which is double that of women (*sr*^2^ = .25). Higher levels of depression accounted for a smaller but still large proportion of variance in self-report of memory problems, with a larger effect for men (*sr*^2^ = .30) than for women (*sr*^2^ = .15).

### Influence of manganese exposure

To examine the influence of Mn levels on the self-report of concentration and memory problems, a similar hierarchical multiple linear regression was conducted. Age, sex, years of education, household income, depression, and anxiety were entered as covariates in Step 1 of the model. Measures of Mn in the air and blood did not account for the variance in the scaled report of concentration and memory problems (data not shown). Distance from the Mn source accounts for 4% of the total variance in the self-report of memory problems, but depression remains the largest predictor, accounting for 21% of the total variance.

## Discussion

Few previous studies have examined the validity of self-reported concentration and memory problems in middle-aged adults using multiple self-report metrics. Consistent with previous research, the present study found no or only weak relationships between self-report of concentration and memory problems and neuropsychological test scores. This finding is consistent with prior neuropsychological research (McConnell & Crockett, 1994). Although neither metric demonstrated adequate convergent validity, scaled items appeared to be more sensitive than dichotomized items when measuring self-reported cognitive dysfunction.

In our sample, 73.3 and 65.3% of participants reported problems concentrating and remembering, respectively. When compared to a demographically similar exposed sample from another epidemiological study (Bowler et al., 1996), 78.6 and 76.6% of exposed participants reported problems concentrating and remembering, respectively. In comparison, 32.9 and 40.5% of unexposed control participants reported problems concentrating and remembering, respectively. In another epidemiological study, 41% of unexposed control participants reported concentration or memory problems (Bowler et al., 1997). As expected, in an exposed sample, the proportion of participants who perceive concentration or memory problems is higher than in the unexposed population. We propose that this perception of cognitive problems is largely related to symptoms of depression.

The literature strongly supports the notion that the severity of subjective cognitive complaints is influenced by depressive and anxious symptoms or psychological distress (Chin et al., 2014; Heaton & Pendleton, 1981; Iliffe & Pealing, 2010; Jungwirth et al., 2004; Langlois & Belleville, 2014; Larrabee & Levin, 1986; McConnell & Crockett, 1994). In some studies, the relationship between subjective and objective cognitive assessment became substantially weaker when the effects of depression were accounted for (Crumley et al., 2014; Genziani et al., 2013). Depression was found to be the strongest predictor associated with greater self-report of concentration and memory problems. Residents with lower education, those with higher levels of anxiety, and women reported more concentration and memory problems. Levels of Mn were not related to subjective concentration and memory problems.

One possible explanation for the finding that depression predicts self-reported cognitive problems in both men and women may be that depressed persons negatively evaluate benign lapses in memory as being indicative of more serious cognitive impairment (Chin et al., 2014; McConnell & Crockett, 1994). A ruminative personality style, low self-esteem, and greater self-focused attention are commonly associated with increased depressive symptoms (American Psychiatric Association, 2013; Takano & Tanno, 2009). These traits have also been shown to predict greater subjective cognitive complaints (Chin et al., 2014; Uttl & Kibreab, 2011). Increased focus on one's own cognitive abilities combined with rumination over normal memory lapses may lead to the misperception that one's cognitive abilities are impaired.

It is not clear why self-report of concentration and memory problems are more common in women than in men. We analyzed numerous mood factors, including all subscales of the SCL-90-R (e.g., Somatization), sociodemographic characteristics (i.e., age, education, ethnicity, personal and household income, marital status, employment), and Mn exposure measures, but no explanation can be offered for this sex difference in self-report of concentration and memory problems. It was determined, however, that a higher level of depression was a larger predictor of self-report of concentration and memory problems in men than in women. Specifically, higher levels of depression accounted for twice the amount of variance in both self-reported concentration and memory problems for men than for women. Future research is needed to explain these gender differences when measuring self-report of cognitive problems.

Crumley et al. (2014), in their meta-analysis of 53 studies of older adults, found a stronger correlation between self-report and objective assessment of memory in women whereas other studies report smaller correlations for women than for men (Hülür et al., 2014; Volz-Sidiropoulou & Gauggel, 2012).

Although distance from the Mn source, the participants' residence, and their modeled Mn level accounted for a small amount (4%) of the total variance in the residents' self-report of memory and concentration problems, depression far outweighs the impact of Mn exposure on the residents' self-perception of having cognitive problems.

In summary, our findings indicate that self-reports of concentration and memory problems are not valid indicators of cognitive function, and appear to reflect higher levels of depression.

### Strengths

A strength of this study is the availability of sensitive and comprehensive neuropsychological screening tests administered by highly trained psychometricians. Additionally, participants' scores on all neuropsychological tests were standardized using age-adjusted normative data. This standardization addresses normal age-related variability in the function assessed by each test.

Additional strengths of this study include the strict exclusion criteria and the restricted age range of 30–64 years. This resulted in a study sample of middle-aged adults without known neurodegenerative disorders, which provides a contribution to the relatively small body of literature examining the validity of self-reported concentration and memory problems.

In addition, the possibility of a selection bias was minimized as demonstrated by the fact that both Marietta and East Liverpool participants were mostly representative of the 2008–2012 U.S. Census data for the two respective towns (Bowler, Beseler, et al., 2016).

### Limitations

Despite the use of the SCL-90-R, a limitation of this study is the lack of personality measures to determine participants' long-term personality styles and how these characteristics might influence participants' subjective beliefs about their cognitive abilities. Understanding these personality variables might help to further disentangle the mechanisms by which depression and anxiety influence subjective beliefs about cognitive impairment.

Additionally, data collection from the two Ohio towns was not completed at the same time. Because of funding limitations, data from East Liverpool were not collected until 2011 (data from Marietta were collected in 2009). However, the strict inclusion criteria and identical test administration procedures served to mitigate this limitation. No significant regional events occurred between 2009 and 2011 that would have altered participants' assessments.

Although the Rey 15-Item Memory Test is a popular measure of participant effort, some studies have found this measure to lack sufficient sensitivity when identifying malingering (Lezak et al., 2012). For this reason, in the current study, the Rey 15-Item Memory Test was used to screen participants for insufficient effort. If malingering was suspected, the more sensitive VSVT was administered as well as a clinical interview with the principal investigator, who is an experienced clinical neuropsychologist.

## Conclusion

This study supports and expands the knowledge that not only older populations, but also groups of middle-aged persons, appear to misperceive their actual levels of cognitive dysfunction as measured by clinical neuropsychological assessment. When only self-report of concentration and memory problems are used without neuropsychological assessment validation, the presence of cognitive dysfunction is likely misrepresented or inflated. As shown here, self-report of cognitive problems should be further investigated in health studies as it is likely associated with mood problems such as depression. Although mild cognitive deficits were associated with air-Mn (Bowler et al., 2015), no differences in neuropsychological performance were observed between those residents who self-reported cognitive problems and those residents whodid not. Misperceptions of cognitive dysfunction appear to be the result of greater levels of depression, which may subsequently compound well-being and result in increased report of cognitive impairment. Identifying objective cognitive dysfunction with neuropsychological assessment also permits appropriate treatment recommendations, which should include cognitive rehabilitation. In conclusion, caution is recommended when interpreting self-reports of cognitive problems in community residents as synonymous with clinical impairment.

## Figures and Tables

**Table 1:** Neuropsychological test battery.

Domains of function and tests administered	Cognitive function(s) assessed	Type of score
*Concentration*		
Trails A	Visual scanning and visuomotor tracking of sequential numbers	*T* score^a^
Trails B	Category switching, sequencing, scanning, sustained concentration	*T* score^a^
WAIS–III Digit Span	Attention and working memory	Scaled score^b^
WAIS–III Digit Symbol Coding	Fine visual–motor speed and accuracy of nonverbal learning	Scaled score^b^
WAIS–III Similarities	Capacity for verbal concept formation, abstract thinking	Scaled score^b^
*Memory*		
Auditory Consonant Trigrams Mean	Measure of frontal lobe function, memory	*z*-score^c^
NAB Memory Index	Overall performance on visual and verbal immediate and delayed memory	Standard score^d^
Rey–Osterrieth Delayed Recall	Visuospatial constructional ability and delayed (30 min) recall	*T* score^e^

Note. WAIS–III = Wechsler Adult Intelligence Scale–Third Edition. *T* score: *M* = 50, *SD* = 10. Scaled score: *M* = 10, *SD* = 3. *z* score: *M* = 0, *SD* = 1. Standard, score: *M* = 100, *SD* = 15

a Age, gender, education, and ethnicity corrected (Heaton et al., 2004)

b Age corrected (Wechsler, 1997).

c Age corrected (Boone et al., 1990).

d Age, gender, and education corrected (Stern & White, 2003).

e Age corrected (Strauss et al., 2006).

**Table 2:** Sociodemographics, exposure, and cognitive complaints.

Characteristic	*n*	*M ± SD*	%
*Continuous*			
Age	146	51.24 ± 8.6	
Years of education	146	13.88 ± 2.4	
Years of residence	146	38.58 ± 15.8	
Distance from Mn source (air miles)	146	3.01 ± 2.1	
Air manganese (μgm^−3^)	146	0.55 ± 0.9	
Blood manganese (μg L^−1^)	146	10.13 ± 3.5	
*Categorical*			
*Sex*			
Male	60		41.1
Female	86		58.9
*Race*			
White	138		94.5
Non-White	8		5.5
*Annual household income*^a^			
$0–29,999	42		30.2
$30,000–69,999	51		36.7
$70,000+	46		33.1
*Trouble concentrating (HQ)*^b^			
Yes	39		27.1
No	105		72.9
*Trouble remembering (HQ)*			
Yes	66		45.2
No	80		54.8
*Trouble concentrating (SCL-90-R)*			
Not at all	52		35.6
A little	54		37.0
Moderately	22		15.1
Quite a bit	14		9.6
Extremely	4		2.7
*Trouble remembering (SCL-90-R)*			
Not at all	41		28.1
A little	54		37.0
Moderately	19		13.0
Quite a bit	22		15.1
Extremely	10		6.8

Note. HQ = health questionnaire; SCL-90-R = Symptom Checklist-90-Revised.

a *n* = 139.

b *n* = 144.

**Table 3:** Bivariate correlation coefficients between subjective self-report and objective neuropsychological test performance.

Domain	Neuropsychological test	Concentration problems		Memory problems	
			
		Dichotomized	Scaled	Dichotomized	Scaled
Concentration	Trails A	−.075^a^	−.065	.014	−.038
	Trails B	−.115^b^	−.157^c^	−.070^c^	−.126^c^
	WAIS Digit Span	.024^a^	−.085	−.037	−.141
	WAIS Digit Symbol Coding	−.093^a^	−.184	−.111	−.170
	WAIS Similarities	−.122^a^	−.180	−.133	−.147
Memory	ACT Mean	−.073^b^	−.103^c^	−.196^c^	−.180^c^
	NAB Memory Index	.040^a^	−.042	.022	.007
	Rey–O Delayed Recall	−.108^a^	−.175	−.165	−.198

Note. Benjamini–Hochberg corrected false discovery rate probabilities used. WAIS = Wechsler Adult Intelligence Scale; ACT = Auditory Consonant Trigrams; NAB = Neuropsychological Assessment Battery; Rey–O = Rey−Osterrieth.

a *n* = 144.

b *n* = 143.

c *n* = 145.

**Table 4:** Binomial logistic regression results of anxiety and/or depression predicting dichotomized self-reported concentration and memory problems.

Self-report item	Step	Variable entered	*B*	*OR*	*p*
Concentration problems (Yes/No)^a^	1	Age	0.016	1.02	.535
		Sex	−0.953	0.39	.059
		Education	−0.135	0.87	.230
		Income	0.133	1.14	.159
	2	Res. anxiety	0.006	1.01	.828
		Depression	0.116	1.12	**<.0001**
Memory problems (Yes/No)^b^	1	Age	0.001	1.00	.966
		Sex	−0.982	0.37	.021
		Education	−0.208	0.81	.030
		Income	0.091	1.10	.249
	2	Res. anxiety	0.015	1.02	.586
		Depression	0.088	1.09	**<.0001**

Note. Res. anxiety = residualized anxiety; *OR* = odds ratio; values in boldface indicate *p* < .05.

a *n* = 137.

b *n* = 139.

**Table 5:** Hierarchical linear regression results of anxiety and/or, depression predicting scaled self-reported concentration and, memory problems.

Self-report item	Step	Variable entered	*B*	*sr*^2^	*p*
Concentration problems (0–4)^a^	1	Age	0.014	.00	.834
		Sex	−0.167	.03	**.016**
		Education	−0.099	.01	.187
		Income	0.105	.01	.191
	2	Res. anxiety	0.160	.02	**.020**
		Depression	0.648	.39	**<.0001**
Memory problems (0–4)^a^	1	Age	0.088	.01	.252
		Sex	−0.196	.03	**.013**
		Education	−0.133	.01	.123
		Income	0.045	.00	.623
	2	Res. anxiety	0.030	.00	.696
		Depression	0.470	.20	**<.0001**

Note. Res. anxiety = residualized anxiety; values in boldface indicate *p* < .05.

a *n* = 138.
